# DAILY DYADIC COPING AND SALIVARY CORTISOL RHYTHMS IN COUPLES LIVING WITH EARLY-STAGE DEMENTIA

**DOI:** 10.1093/geroni/igae098.4149

**Published:** 2024-12-31

**Authors:** Courtney Polenick, Angela Turkelson, Charity Garner, Yin Liu, Kira Birditt

**Affiliations:** University of Michigan, Ann Arbor, Michigan, United States; University of Michigan, Ann Arbor, Michigan, United States; University of Michigan, Ann Arbor, Michigan, United States; Utah State University, Logan, Utah, United States; University of Michigan, Ann Arbor, Michigan, United States

## Abstract

Dyadic coping (i.e., how couples cope with stress) is associated with subjective well-being in dementia care dyads. However, we know little about dyadic coping and physiological stress reactivity in the context of daily dementia care. We evaluated how daily perceptions of individual and partner supportive (when one partner provides problem-focused and/or emotion-focused coping to help the other) and delegated (when one partner takes over responsibilities to help the other) dyadic coping are associated with salivary cortisol among couples living with early-stage dementia. Participants included 34 people living with dementia (PLWD; M = 72.2 years, SD = 7.5) and their spouse caregivers (M = 69.5 years, SD = 8.7) who completed 7 days of morning and evening phone interviews and collected saliva samples (four times per day) during four of those days. We estimated multilevel models with outcomes including the cortisol awakening response (AR; awakening to 30-min post-awakening) and wake-evening slope (WES; 30-min post-awakening to bedtime). PLWD had a higher next-day AR when they reported higher individual supportive and delegated dyadic coping. PLWD also had a higher same-day and next-day AR and a steeper decline in WES when they reported higher partner delegated dyadic coping. On average across days, PLWD had a flatter decline in WES when caregivers reported higher partner delegated dyadic coping. Finally, on average across days, caregivers had a higher AR when they reported higher individual supportive and delegated dyadic coping. These findings identify targeted strategies to improve health and well-being in couples navigating early-stage dementia.

